# Case report of a primary subcutaneous melanoma; a surprising entity for a subcutaneous nodule

**DOI:** 10.1016/j.ijscr.2021.106359

**Published:** 2021-08-31

**Authors:** E. Jutten, M.F. Lutke Holzik, A. Baidoshvili, R.R. Dulfer

**Affiliations:** a University Medical Center Groningen, Hanzeplein 1, 9713 GZ, Groningen, the Netherlands; b Hospital Group Twente, Zilvermeeuw 1, 7609 PP Almelo, the Netherlands; c Laboratory for Pathology East Netherlands, Boerhaavelaan 59, 7555 BB Hengelo, the Netherlands

**Keywords:** Case report, Primary dermal melanoma, PDM, Subcutaneous melanoma

## Abstract

**Introduction:**

A melanoma can originate at the subcutis without any visible skin lesion.

**Case presentation:**

A 73-year old patient came to the outpatient clinic with a subcutaneous nodule on the right thigh without any visible lesion of the skin. It turned out to be a primary subcutaneous melanoma that could be classified as a primary dermal melanoma (PDM).

**Discussion:**

A PDM is a very rare subtype of melanoma that stands out for its excellent prognosis in comparison to cutaneous melanomas. No valid reliable staging system or treatment guideline exists for this entity, Breslow depth might overestimate the clinical aggressiveness possibly leading to overtreatment.

**Conclusion:**

It is of great importance for the clinician to be familiar with a primary dermal melanoma. It deserves an appropriate place in the current AJCC system and a treatment guideline for this unique melanoma subtype with relativity excellent prognosis would be beneficial.

## Highlights

• A melanoma can originate at the subcutis without any visible skin lesion.
• This entity can be classified as a primary dermal melanoma and stands out for its excellent prognosis.
• There is no adequate staging system yet for patients with a primary dermal melanoma.
• Breslow depth might overestimate the clinical aggressiveness in patients with a primary dermal melanoma, possibly leading to overtreatment.

## 1 Introduction

In this paper, we describe a case of a primary subcutaneous melanoma; a surprising entity for a subcutaneous nodule. It seems this entity can be classified as a primary dermal melanoma (PDM). This is a very rare subtype of cutaneous melanoma which has no epidermal component but is localized to the dermis or subcutaneous tissue. This melanoma subtype is rarely reported in the literature, but stands out for its excellent prognosis in comparison to cutaneous melanomas.

A primary dermal melanoma can be defined by the presence of an isolated dermal or subcutaneous melanoma without evidence of an associated nevoid precursor, follicular epithelium connection, or a dermal-epidermal junctional component [1], [2]. There must be absence of an in situ component, surface ulcerations, evidence of overlying regression or scaring [1]. The possibility of metastasis from cutaneous or occult extracutaneous sites must be excluded [1], [3].

The available literature suggests a remarkably good long-term survival of a PDM in comparison with similarly staged conventional cutaneous melanomas; the 5-year survival rate of PDM is 73–100% [2], [4], [5], [6], [7] against a 5–19% 5-year survival for epidermal involved melanoma with similar Breslow thickness [4], [7]. Therefore it is of great importance for the clinician to be familiar with the possibility a subcutaneous tumor can be a primary dermal melanoma, and it would be beneficial that this unique melanoma subtype with relatively excellent prognosis will be appointed in the current melanoma treatment guidelines and AJCC staging system.

This work has been reported in line with the SCARE 2020 criteria [8].

## 2 Presentation of case

### 2.1 Patient information and clinical findings

In July 2020, a 73-year old patient came to the outpatient clinic of a community hospital with a subcutaneous nodule on the right thigh, without any visible lesion of the skin. The medical history included hypertension, atrial fibrillation, a cerebrovascular accident, aortic valve replacement, total knee replacement, Parkinson's disease and recently a radically removed squamous cell carcinoma on the forehead. The patient was wheelchair-dependent and living in a nursing home.

The subcutaneous nodule existed on presentation for about a year and was sometimes irritating. On physical examination a 4 cm firm elastic nodule was found, no sinus was present. The differential diagnosis at that time included lipoma or sarcoma.

### 2.2 Diagnostic assessment and therapeutic intervention

**Fig. 1:**
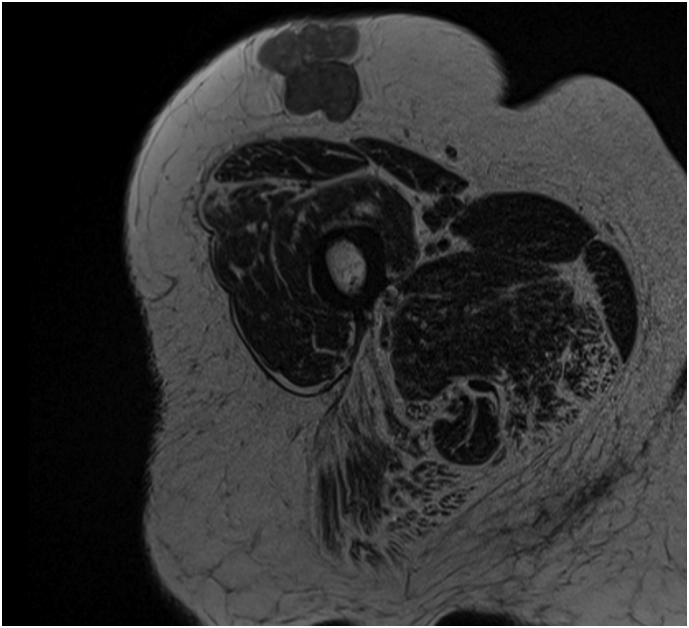
MRI image of the subcutaneous lesion.

Since the nature of the lesion and relation to the underlying surface could not be judged properly an MRI was made. The MRI showed a 44 mm non-specific subcutaneous lesion (Fig. 1) and was thought to be a metastasis or primary neoplasm. From this moment, every step in the process was discussed in a multidisciplinary team.

**Fig. 2:**
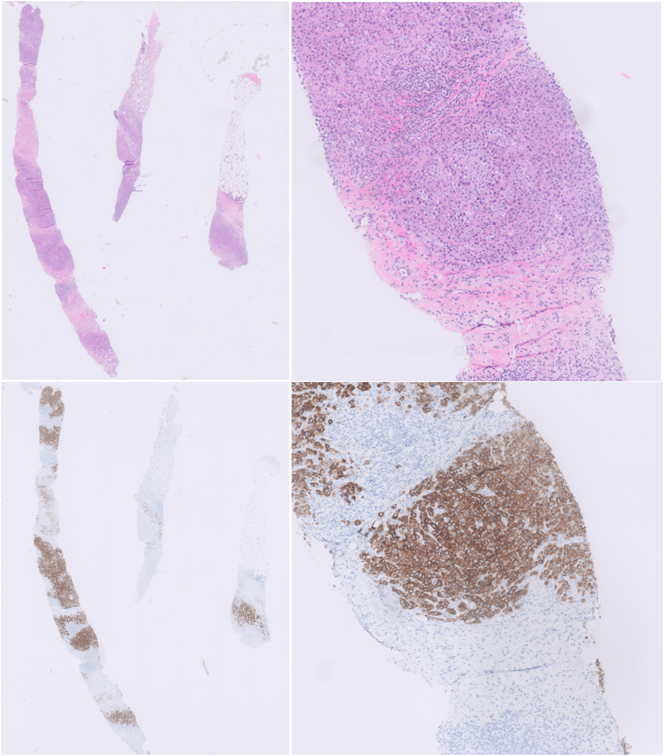
Needle biopsy with clusters of atypical melanocytic cells (Haematoxylin Eosin and Melan-A, magnification 0,5× and 5×).

First an ultrasound-guided needle biopsy was performed and the histopathological report concluded malignant melanoma (clusters of atypical cells with variable hyperchromic and prominent nuclei, relatively eosinophilic cytoplasm, SOX10: positive, Melan-A: positive, HMB-45: negative to weak positive, AE1/AE3: negative to weak positive, P40: negative (Fig. 2)). Laboratory results showed S100: 0.04, LD: 189.

Differential diagnostic considerations were this could be a primary or metastatic (cutaneous or non-cutaneous) melanoma. To identify the kind of this melanoma additional clinical examination and imaging was conducted. To exclude a possible other cutaneous primary melanoma lesion a total body clinical examination by a dermatologist was performed. The dermatologist did found some seborrheic keratosis but no suspect nevi as a primary tumor. To identify a possible other non-cutaneous or metastatic melanoma a total body PET-CT was performed. The PET-CT showed a high FDG-uptake at the subcutaneous lesion on the right thigh and high muscle activity around the left supraspinatus muscle. There was no involvement of lymph nodes and there were no other suspect spots. Thoughts were the high muscle activity around the left supraspinatus muscle could be a metastasis although it was not a typical presentation. An ultrasound-guided needle biopsy of the left supraspinatus muscle was planned, but the ultrasound showed only degenerative changes of the rotator cuff and no focal lesions in the supraspinatus muscle. Therefore, no biopsy was performed. Hence, it became much more likely the subcutaneous nodule was a primary subcutaneous melanoma.

**Fig. 3:**
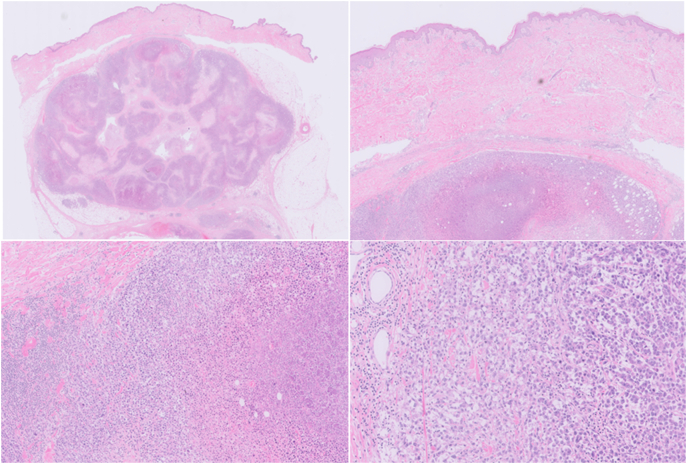
Histology of the primary dermal melanoma (Haematoxylin Eosin, magnification 0,25×, 1×, 2× and 10×).

A wide excision of the subcutaneous lesion on the right thigh was performed by the surgeon. Considering the abovementioned medical comorbidity of the patient, and considering the PET-CT did not show inguinal nodal involvement, a sentinel node procedure was not performed. The pathological report concluded a melanoma of 39 mm, Breslow thickness 35,9 mm, no ulceration, no microsatellites, no regression and clear margins (Fig. 3). 8th AJCC TNM classification: pT4a. To be absolutely sure to differentiate with clear cell sarcoma a molecular analysis could have been performed. However, due to the medical comorbidity of the patient (the patient would not have been eligible for systemic therapy) this was not performed since there would be no consequences.

### 2.3 Follow-up and outcomes

Due to the previously mentioned medical comorbidity of this patient it was decided in multidisciplinary team to opt for an expectant policy and clinical follow-up. Follow-up was planned to consist of a three-monthly outpatient clinic visit, alternated at the dermatologist and surgeon for the first 2 years. Three months after surgery, there were no complications and patient was doing well. Unfortunately, 4 months after surgery the patient died in the nursing home due to a cause unrelated to this melanoma.

## 3 Discussion

A primary subcutaneous melanoma or any other PDM is a rare phenomenon. There are some previous studies; a few case reports and single center series who have identified subcutaneous melanomas with no known separate primary melanoma and negative metastatic findings on work-up. In 2004, Swetter et al. [2] proposed PDM as a distinct entity based on an excellent prognosis. They found 7 patients with a dermal or subcutaneous melanoma without a junctional or epidermal component with a much better prognosis (100% survival after a mean follow-up of 41 months) in comparison to epidermal involved melanoma with similar Breslow thickness. After this study Cassarino et al. [7], Sidiropoulos et al. [4], Teow et al. [1], and Harris et al. [6] likewise described patients with a dermal or subcutaneous melanoma with an excellent prognosis.

Meanwhile, several theories have been proposed regarding the pathogenesis of PDM. A plausible explanation is that the tumor actually originate in the dermal and/or subcutaneous tissue, possibly from nonepidermal melanocytes, embryologic-melanocytic migration remnants or aberrations, or melanocytes associated with deeper appendageal structures [3]. Other authors believed that spontaneous regression of the primary cutaneous lesion was the explanation [9], that spontaneous loss of the overlying epidermal component due to trauma, biopsy, scarring following prior treatment or inflammation was the explanation [6], or that PDM's were in fact dermal metastases [4]. However, due to the survival data which are far too favorable in patients with PDM to align with metastatic disease, this last theory is highly unlikely. To date, the definition by Teow et al. [1] for PDM stated there must be absence of an in situ component, surface ulceration, evidence of overlying regression or scarring.

Currently, no valid reliable staging system exists for PDM. In the 6th edition (2002) of the American Joint Committee on Cancer (AJCC) staging systems, a PDM was considered to be a metastasis from an unknown site and therefore classified as stage IV M1a disease, which represents an extremely poor prognosis, namely a 5-year survival rate of 18% [10]. In the 7th AJCC edition (2009) this melanoma was categorized as stage III, reflecting the better prognosis of this patients and doing justice to the possibility that some cases might indeed represent a primary lesion [11]. Recently, the AJCC has been updated but does not include PDM [12]. In the current AJCC staging system, the major prognosticators for melanomas are Breslow depth, Clark level, mitotic rate, ulceration and sentinel node status [12]. These factors may fall short of prognosticating patients with PDM. Likewise our patient is classified as pT4a, not doing justice to the prognosis of this disease, and - if the patient was in a better condition - possibly leading to overtreatment.

It is likely that traditional Breslow depth measurement overestimates the clinical aggressiveness in patients with a primary subcutaneous melanoma since it does originate at a subcutaneous level instead of cutaneous. A suggestion made by Swetter et al. [2] we therefore endorse is to measure the maximum tumor diameter in a vertical plane to better reflect the outcome.

To date, there is no treatment guideline for PDM and the situation that there is no appropriate staging system creates a significant therapeutic dilemma whether to treat the patient as a real pT4a or not. Authors differ in their treatment recommendations regarding the need for sentinel node biopsy, adjuvant therapy and follow up [1], [5], [13]. Since patients with PDM has shown favorable prognosis with surgery alone this might be sufficient [13].

## 4 Conclusion

It is of great importance for the clinician to be familiar with a primary dermal melanoma. There is no adequate staging system yet; Breslow depth might overestimate the clinical aggressiveness possibly leading to overtreatment. A treatment guideline for this unique melanoma subtype with relativity excellent prognosis would be beneficial.

## Provenance and peer review

Not commissioned, externally peer-reviewed.

## Sources of funding

E. Jutten received a grant from the Anna Dorothea Hingst Stichting and the Groningen Melanoma Sarcoma Foundation. (Both did not have any role in study design; in the collection, analysis and interpretation of data; in the writing of the report; or in the decision to submit the article for publication.)

## Ethical approval

This case report is exempt from ethical approval.

## Consent

Written informed consent was obtained from the patient next of kin for publication of this case report and accompanying images. A copy of the written consent is available for review by the Editor-in-Chief of this journal on request.

## Research registration (for case reports detailing a new surgical technique or new equipment/technology)

N/A.

## Guarantor

E. Jutten

## CRediT authorship contribution statement

E. Jutten: Conceptualization, writing – original draft, writing – review and editing.

M.F. Lutke Holzik: Conceptualization, supervision, writing – review and editing.

A. Baidoshvili: Acquisition of data, analysis and interpretation of data, supervision, writing – review and editing.

R.R. Dulfer: Conceptualization, supervision, writing – review and editing.

## Declaration of competing interest

None.
